# The process of mammalian mitochondrial protein synthesis

**DOI:** 10.1007/s00441-016-2456-0

**Published:** 2016-07-14

**Authors:** Nicole Mai, Zofia M. A. Chrzanowska-Lightowlers, Robert N. Lightowlers

**Affiliations:** 1 Wellcome Trust Centre for Mitochondrial Research, Institute of Neuroscience, Medical School, Newcastle University, Newcastle upon Tyne, NE2 4HH UK; 2 Wellcome Trust Centre for Mitochondrial Research, Institute for Cell and Molecular Biosciences, Medical School, Newcastle University, Newcastle upon Tyne, NE2 4HH UK

**Keywords:** Mitochondria, Mitoribosome, Mitochondrial translation, Protein synthesis, Mitochondrial diseases

## Abstract

Oxidative phosphorylation (OXPHOS) is the mechanism whereby ATP, the major energy source for the cell, is produced by harnessing cellular respiration in the mitochondrion. This is facilitated by five multi-subunit complexes housed within the inner mitochondrial membrane. These complexes, with the exception of complex II, are of a dual genetic origin, requiring expression from nuclear and mitochondrial genes. Mitochondrially encoded mRNA is translated on the mitochondrial ribosome (mitoribosome) and the recent release of the near atomic resolution structure of the mammalian mitoribosome has highlighted its peculiar features. However, whereas some aspects of mitochondrial translation are understood, much is to be learnt about the presentation of mitochondrial mRNA to the mitoribosome, the biogenesis of the machinery, the exact role of the membrane, the constitution of the translocon/insertion machinery and the regulation of translation in the mitochondrion. This review addresses our current knowledge of mammalian mitochondrial gene expression, highlights key questions and indicates how defects in this process can result in profound mitochondrial disease.

## Introduction

Mitochondria are organelles that perform several different functions crucial for the homeostasis of most eukaryotic cells. The suggestion has been made that these double-layered organelles derive from the endocytosis of an α-proteobacterium by a pre-eukaryotic cell (Gray 1999; Falkenberg et al. 2007) and that the bacterium was retained as it conferred a selective advantage through the production of ATP. This molecule provides chemical energy for cells and is a product of oxidative phosphorylation (OXPHOS), which takes place at the inner mitochondrial membrane (IMM). Oxidative phosphorylation utilises five multi-subunit complexes, of which four contain a unique combination of both nuclear and mitochondrial (mt) DNA-encoded polypeptides. Human mitochondria house multiple copies of a 16.6-kb circular genome, mtDNA, which encodes 13 proteins translated from 11 mt-mRNA species (9 monocistronic and 2 bicistronic units), plus two mt-rRNAs and 22 mt-tRNAs (Anderson et al. 1981). The 13 polypeptides are all components of the OXPHOS machinery and are synthesised within the organelle by using the mitochondrial translation mechanism, the main component being the mitoribosome (O'Brien 1971). Although all RNA components of this particle are mtDNA-encoded, all the 80 or so protein constituents are derived from nuclear genes, as are all the accessory and biogenesis factors involved in intramitochondrial protein synthesis. The nuclear-encoded proteins are synthesised on cytosolic ribosomes, targeted to the mitochondria and imported into the matrix (for a review, see Neupert 2015). The assembled mitoribosome translates the mt-mRNAs, synthesising proteins that are rapidly inserted into the IMM and integrated into their relevant complexes to form the OXPHOS system.

This review aims to summarise the current knowledge of the mammalian mitochondrial translation system including mitoribosomal biogenesis, pre- and post-translational events involving modification, stabilisation and the fate of mitochondrial transcripts. Finally, a brief overview of known pathogenic mutations related to this process will also be presented.

## The mitoribosome

The mitochondrial protein synthesis machinery was first identified by John R. McLean in 1958 in rat liver (McLean et al. 1958) and then isolated from the same organ by Thomas W. O’Brien in 1968 (O'Brien and Kalf 1967). Although mammalian mitoribosomes, like other ribosomes, are composed of a large (mt-LSU) and a small (mt-SSU) subunit, they differ markedly in their density, sedimenting as 55S particles rather than the 80S or 70S species of their eukaryotic cytosolic or bacterial counterparts, respectively (O'Brien 1971). The first cryo-electron-microscopic (cryo-EM) structure of the bovine monosome (the fundamental synthesising particle comprising mt-SSU and mt–LSU) was released in 2003 and showed how significantly the mammalian mitoribosome diverged from its bacterial ancestor (Sharma et al. 2003). The recent release of sub-nanometre resolution structures of porcine (Greber et al. 2014a, 2014b, 2015) and human (Brown et al. 2014; Amunts et al. 2015) mitoribosomes from cryo-EM studies, further highlights these structural and compositional differences (Table 1); an excellent recent review has been published on the structure of the mammalian mitoribosome (Greber and Ban 2016). Whereas bacterial ribosomes are mainly composed of RNA (~70:30% per weight), mammalian mitoribosomes have reversed this ratio and are higher in protein content. This is the result of both the removal of rRNA domains and the increase of the overall protein mass. The increase is effected by the addition of extensions onto many homologous proteins and the acquisition of mitochondrial-specific (mt-specific) proteins, some of which have previously been identified in early proteomics studies (Koc et al. 2001a, 2001b). In a few cases, the new protein mass fills the void generated by the rRNA deletion but, overall, the increase of protein content is mainly found peripherally (Brown et al. 2014) and has been suggested to act as a protective shield for the mt-rRNA, preventing potential damage attributable to the high ROS levels found within the organelle (Lightowlers et al. 2014). The presence of the majority of the mt-specific proteins on the external surface of the subunits suggests that the functional core of the mitoribosome, composed of the mt-mRNA recognition site on the mt-SSU (Amunts et al. 2015; Greber et al. 2015) and peptidyl transferase centre in the mt-LSU (Brown et al. 2014; Greber et al. 2014a, 2014b), has been preserved. This exchange of RNA predominance to protein in the mitoribosome also leads to a different composition of the intersubunit bridges. In bacteria, the two ribosomal subunits interact mainly via RNA:RNA bridges (Liu and Fredrick 2016), whereas in the mammalian 55S particle, a higher proportion of protein-protein and RNA:protein connections is found (Amunts et al. 2015; Greber et al. 2015).

**Table 1:** Characteristics of bacterial and mammalian mitochondrial (*mt*) ribosomes (*SSU* small mt subunit, *LSU* large mt subunit, *nd* not defined)

Properties	Ribosomes		
	Bacterial (*Escherichia coli*)	Porcine mt	Human mt
Sedimentation coefficient	70S	55S	55S
Mass	2.3 MDa	2.7 MDa	nd
RNA : protein	2:1	1:2	1:2
SSU RNA Proteins	30S 16S rRNA 21	28S 12S rRNA 30 (15 mt-specific)	28S 12S rRNA 30 (14 mt-specific)
LSU RNA Proteins	50S 23S rRNA + 5S rRNA 34	39S 16S rRNA + tRNA^Phe^ ~52 (22 mt-specific)	39S 16S rRNA + tRNA^Val^ ~53 (~23 mt-specific)
References	Wittmann 1982; Ramakrishnan and White 1998	Greber et al.2014a, 2014b, 2015	Amunts et al. 2015; Brown et al. 2014

The newly designated ribosomal nomenclature will be used in this review and identifies mt-specific proteins with “m”, universal orthologous proteins with “u” and bacterial-specific orthologues with “b”. This initial letter is followed by “L” for the mt-LSU or “S” for the mt-SSU, which is then followed by an assigned number. In the case of “b” and “u” ribosomal proteins, the letter “m” is also added at the end of the name to distinguish the mitochondrial proteins from the cytosolic ones (Ban et al. 2014).

### Mitoribosomal small subunit (28S)

**Fig. 1:**
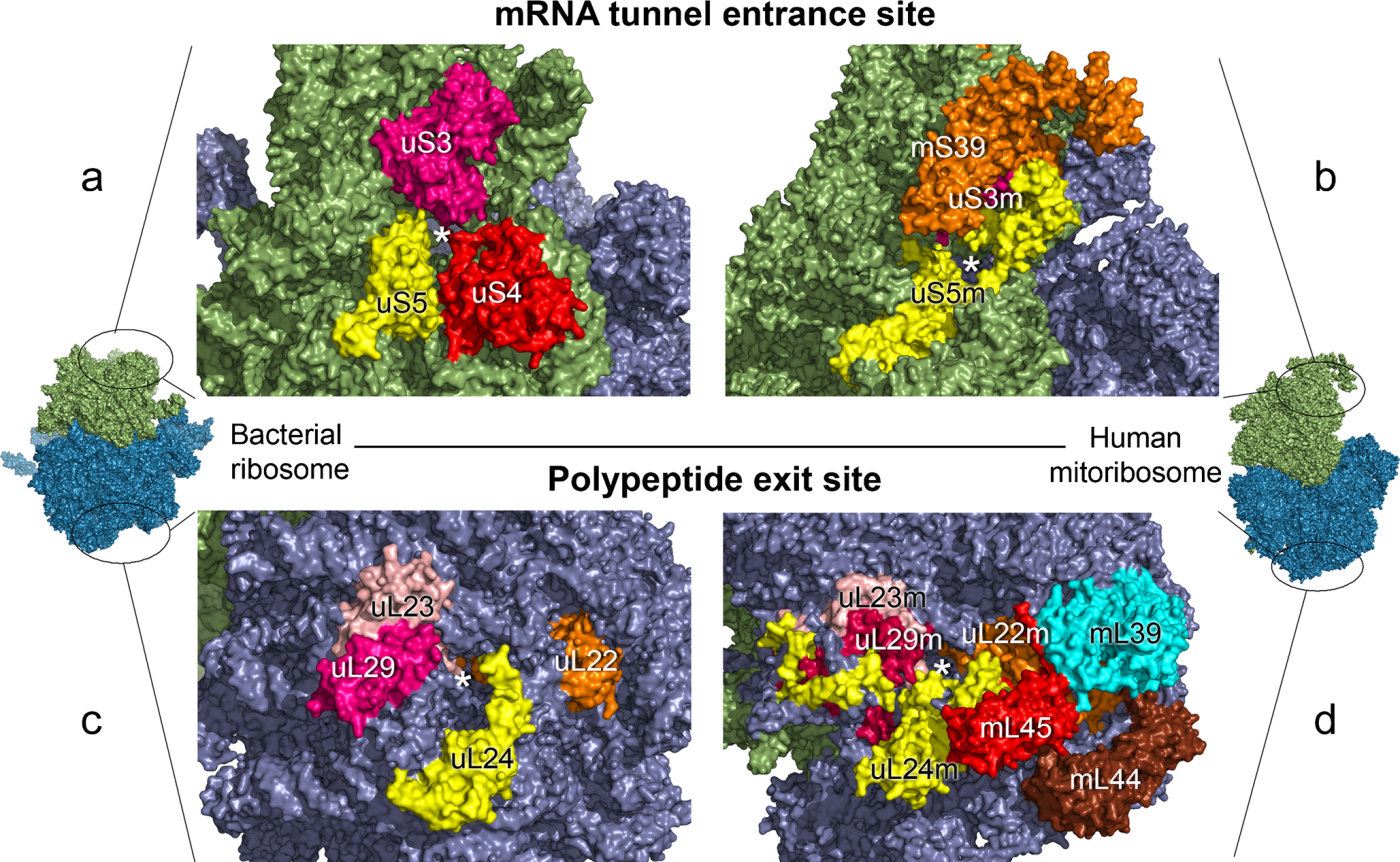
Comparison of structural features of bacterial and human mitochondrial ribosomes. Structures of the *Escherichia coli* ribosome (PDB 4YBB) and the human mitoribosome (PDB 3J9M) were obtained with Pymol (Open Source, Version 1.8.2.0.). The respective monosomes are depicted *left* (*E. coli*) and *right* (*Homo sapiens*) with the location of the entrance to the mRNA tunnel (**a**, **b**) and the exit site of the polypeptide tunnel (**c**, **d**) *circled* to indicate the region expanded in the main part of the figure (*green* small subunit structures, *blue* large subunit structures). Comparison of the entrance to the mRNA tunnel indicates that uS3 (*dark pink*) and uS5 (*yellow*) are present in the ribosomes from both human (**b**) and bacterial **a**) mitochondria. The bacterial entrance site is, in part, defined by uS4 (*red*), whereas in mitoribosomes, an additional RNA-binding protein, mS39 (*orange*), lies close to the entrance and is involved in mRNA recruitment. The bacterial polypeptide exit site (**c**) is defined by uL22 (*orange*), uL23 (*pink*), uL24 (*yellow*) and uL29 (*dark pink*). All of these are present in human mitoribosomes (**d**), with a further ring of proteins surrounding the exit site composed of mL39 (*cyan*), mL44 (*brown*) and mL45 (*red*). Amongst these, mL45 might be involved in anchoring the structure to the inner mitochondrial membrane

To date, the mammalian mt-SSU is thought to be composed of a 12S mt-rRNA and 30 proteins, of which 15 (porcine) or 14 (human) are mt-specific (Amunts et al. 2015; Greber et al. 2015). The increased protein content of the mitoribosome results in a more elongated subunit compared with the shape of the bacterial counterpart. One of the most remodelled areas is the entrance of the mt-mRNA channel. In the bacterial ribosome (Fig. 1a), uS4 and the C-terminus of uS3 are important for defining the ring-shaped entrance of the channel; both are absent in mt-SSU. The mammalian mitoribosomes (Fig. 1b) compensate for this loss by the extension to uS5m, which now defines the entrance of the channel. The mt-specific protein, mS39 (PTCD3), also lies in close proximity to this channel. PTCD3 is a member of the pentatricopeptide-repeat (PPR)-containing protein family, characterised by their RNA-binding ability (Filipovska and Rackham 2013; Lightowlers and Chrzanowska-Lightowlers 2013). This feature combined with the location of mS39 suggests that it is involved in the recruitment of mt-mRNA to the monosome. One domain of the 12S mt-rRNA that has been deleted is the anti-Shine-Dalgarno sequence, which would conventionally be located close to the exit of the mRNA channel. The absence of this domain is consistent with and perhaps reflects the absence of the corresponding 5’-untranslated region (UTR) on mt-mRNAs (Montoya et al. 1981). The space generated by the lack of this rRNA domain is now occupied by the mitochondrial-specific protein mS37, which takes on the interaction with the 12S mt-rRNA. In contrast to these structural modifications, the central portion of the mRNA channel, which is directly involved in the translation process, is mostly conserved (Greber et al. 2014a, 2014b).

### Mitoribosomal large subunit (39S)

As with the mt-SSU, several differences can be seen between mt-LSU and its bacterial counterpart. The mammalian mt-LSU is composed of 16S mt-rRNA and the recent ribosomal nomenclature lists 52 proteins (53 in humans), of which 22 are mt-specific proteins (Brown et al. 2014; Greber et al. 2014a, 2014b). With respect to the RNA content, the bacterial LSU contains the 23S and the 5S rRNA, located in the central protuberance (Ban et al. 2000). Intriguingly, the cryo-EM structure of the mammalian mitoribosome reported in 2014 has revealed an RNA density that resembles a domain of bacterial 5S rRNA (Greber et al. 2014a, 2014b) but the authors have been unable to identify its nature. More recent cryo-EM studies of porcine and human mt-LSU particles have confirmed the presence of an additional RNA species and identified this as mt-tRNA^Phe^ (Greber et al. 2014a, 2014b) and mt-tRNA^Val^ (Brown et al. 2014), respectively. Intriguingly, these two mt-tRNA genes are found in close apposition to the 12S and 16S mt-rRNA genes and are transcribed as a single polycistronic RNA unit in mammalian mitochondria. Whereas the mt-LSU from all mammalian species analysed contain only one or other of these mt-tRNA species (and not the other 20 species), no evidence has been obtained for tissue-specific variation. Further, under conditions of mt-tRNA^Val^ depletion caused by a destabilising pathogenic mutation, the human mitoribosome has recently been shown to be able to switch to the incorporation of mt-tRNA^Phe^ and is still functional (J. Rorbach et al., in preparation). One of the areas of the mitoribosome that has changed the most throughout evolution is the polypeptide exit site (PES; Fig. 1c, d). The exit region of the tunnel is defined by a universally conserved ring of proteins (uL22m, uL23m, uL24m) but, in mammals, a second layer of additional mt-specific proteins (mL39, mL44, mL45) are found extending the conserved ring (Fig. 1d). The recruitment of these additional proteins around the exit site might be linked to the synthesis of highly hydrophobic mt-encoded proteins (see Localisation of mitochondrial translation).

### Mitoribosomal A-, P- and E-sites

As described earlier, the functional core of the mitoribosome has been conserved throughout evolution. Similarly to most ribosomes, mitoribosomal A-, P- and E-sites can be identified (Wettstein and Noll 1965). The A-site residues contributed by the mt-SSU are important for decoding, and the mt-LSU has maintained the ability to mediate the main contact between mt-tRNA and 16S mt-rRNA, although the A-site finger structure is missing as a consequence of a loss during evolution of the specific rRNA domain (Greber et al. 2014a, 2014b, 2015). This loss might be related to the different characteristics of mammalian mt-tRNA, which do not all adopt the typical cloverleaf structure (Suzuki et al. 2011). The P-site is also relatively preserved but the interactions made with the mt-tRNA are stronger than those observed for the bacterial ribosome (Schuwirth et al. 2005), as the mt-LSU has a P-site finger that interacts with the T-loop of mt-tRNA while it is bound to the site. Since this T-loop of mammalian mt-tRNAs is smaller than those in bacterial tRNA, the finger-like structure is probably necessary to maintain the required orientation of the RNA species during peptide bond formation. Finally, the presence of an E-site is controversial, as most of the contact points that exist between the bacterial ribosome and tRNA seem to be lost as a consequence of the shortened mt-rRNA. However, the existence of this site has been confirmed in recent cryo-EM studies that have highlighted the presence of a modified binding pocket (Greber et al. 2014a, 2014b).

### Additional roles of mt-specific proteins

Some of the mt-specific mitoribosomal proteins have been suggested to have additional roles. Examples include mS29 (DAP3; Kissil et al. 1999), mL37 (Levshenkova et al. 2004), mL41 (Yoo et al. 2005) and mL65 (previously named mS30; Sun et al. 1998), all of which have been linked to the control of apoptosis, whereas bL12m has been implicated in POLRMT function and stability (Surovtseva et al. 2011; Nouws et al. 2016). In addition to a potential role in apoptosis, mS29 has an intrinsic GTP-binding site and is phosphorylated, which may affect monosome formation since its location is at the interface (Amunts et al. 2015; Greber et al. 2015). The cryo-EM studies performed on human and porcine samples have also confirmed the presence of a ribosome-dependent peptidyl-tRNA hydrolase, ICT1, as a structural component of the mt-LSU (Richter et al. 2010). Although levels of free ICT1 in the mitochondrion are reported to be extremely low (Richter et al. 2010; Chrzanowska-Lightowlers and Lightowlers 2015), ICT1 might function to help release stalled mitoribosomes (Akabane et al. 2014; Feaga et al. 2016). One particularly striking additional role for a mitoribosomal protein concerns uL18m (Zhang et al. 2015). Under conditions of heat shock, a cytosolic isoform of this protein is generated through translational initiation at an internal CUG codon of its cognate transcript. This isoform is integrated into cytosolic 80S ribosomes, which selectively translate mRNAs encoding heat shock proteins. This is a unique example of the way that a mitoribosomal protein can influence stress adaptation in the eukaryotic cell. A role for the same protein, uL18M, has been suggested for 5S RNA import into mitochondria (Smirnov et al. 2011) but no potential function of 5S RNA in mitochondria has yet been determined.

## Pre-translation events

Mitoribosomal biogenesis and processing and the maturation of the mt-transcripts are all prerequisites for intraorganellar protein synthesis. The assembly pathway of the mitoribosomal subunits, however, and the mechanism of recruitment of mt-mRNAs to the mitoribosome remain largely uncharacterised.

### Mt-mRNA processing and stabilisation

All the mt-mRNA transcripts, with the exception of *MTND6*, are matured by a poly(A) polymerase (mtPAP; Tomecki et al. 2004; Slomovic et al. 2005). This process introduces a poly- or oligo(A) extension that serves to complete the UAA stop codon in 7 transcripts (Ojala et al. 1981). Unlike the bacterial or cytosolic counterpart, the role that this modification exerts on mt-mRNA stability does not follow a conventional pattern. In the absence of polyadenylation, a consistent effect is seen on the steady state levels of most transcript species, albeit that this may be an increase or decrease. This pattern is observed irrespective of the mechanism by which the poly(A) tail had been removed, although the manner in which this regulation is effected is still unclear (Tomecki et al. 2004; Nagaike et al. 2005; Wilson et al. 2014; Bratic et al. 2016).

The half-lives of mt-mRNAs can be regulated by *cis*-acting elements, as described above, and by *trans*-acting factors. The best characterised mt-mRNA-specific proteins involved in transcript stability are the LRPPRC/SLIRP complex (Sasarman et al. 2010) and FASTKD4, which prevents their degradation (Wolf and Mootha 2014). The only protein-coding transcript derived from the light strand, *MTND6*, interacts with the mitochondrial isoform of FASTK (Jourdain et al. 2015) and GRSF1, which modulates its stability (Antonicka et al. 2013; Jourdain et al. 2013).

### Maturation of mt-tRNAs

Modifications of bacterial tRNAs are introduced to mediate stability and functionality. This is equally true of mammalian mt-tRNA species (Nagaike et al. 2005; Suzuki et al. 2011). The many different modifications have been comprehensively described by Suzuki (2014) and Salinas-Giege et al. (2015). Once the mt-tRNAs have been modified and the CCA added to the 3’ terminus (Nagaike et al. 2001), the transcripts can be charged with their cognate amino acid by the relevant mitochondrial aminoacyl-tRNA synthetase (Diodato et al. 2014) and can participate in the synthesis of mt-encoded proteins.

### Mitoribosomal assembly

Several studies support the hypothesis that the assembly of the mitoribosome takes place in two mitochondrial subcompartments, defined as nucleoids and RNA granules. Nucleoids are centres of mtDNA maintenance, replication and transcription, whereas post-transcriptional RNA processing and maturation occur in the RNA granules. Both the compartments contain mitoribosome assembly factors and proteins involved in RNA stability, plus a number of mitoribosomal proteins (Bogenhagen et al. 2014; Antonicka and Shoubridge 2015). As a consequence, early mitoribosome biogenesis has been suggested to be initiated in the nucleoids and to be completed in the RNA granules. Irrespective, RNA processing and at least the early stages of mitoribosome assembly clearly occur in close proximity to mtDNA, consistent with co-transcriptional processing and even mitoribosomal assembly.

The biogenesis of ribosomes involves several key players that act on rRNA and ribosomal proteins to assemble complete subunits. Relatively few factors have been identified that are necessary for mammalian mitoribosomal assembly compared with the generation of 80S ribosomal particles (Hage and Tollervey 2004) and, although the steps in the process are mostly undefined, an increasing number of crucial factors are emerging. After transcription, the 12S mt-rRNA is processed from the major polycistronic RNA unit and, at some stage in the assembly pathway, is bound at its 3’ terminal helix by an RNA-binding GTPase, ERAL1, which stabilises the mt-rRNA prior to the final maturation of the mt-SSU (Dennerlein et al. 2010; Uchiumi et al. 2010). Both the 12S and 16S mt-rRNA species are known to be modified. The extent of mt-rRNA modification was originally assessed in hamster RNA, where nine modifications were detected (Dubin and Taylor 1978). These included pseudouridylation, base methylation and 2’-O-ribose methylation at conserved sites. A uracil methylation site was also identified on the 12S mt-rRNA but has not yet been confirmed to be present on the human orthologue. Of the five base methylations confirmed in 12S mt-rRNA (Metodiev et al. 2009), the data are consistent with the modifications being performed by TFB1M (adenine; Seidel-Rogol et al. 2002; Metodiev et al. 2009) and NSUN4 (cytosine; Metodiev et al. 2014). The 16S mt-rRNA is also a substrate for modifications including three 2’-O-ribose methylations performed by methyltransferases (MRM1, MRM2, MRM3; Rorbach et al. 2014; Lee and Bogenhagen 2014) and, potentially, one pseudouridylation (Ofengand and Bakin 1997). The latter was not detected in the hamster-derived 16S and the enzyme that could be responsible is still unknown.

The formation of many ribonucleoprotein particles including ribosomes requires GTPases and ATP-dependent RNA helicases, although, to date, fewer than expected have been identified in mammalian mitochondria (for a review, see De Silva et al. 2015). The energy derived from GTP hydrolysis is used to regulate the association or dissociation of proteins or to promote conformational changes. These proteins might also act as placeholders for proteins that will join the immature ribosome at a later stage in the assembly process. Finally, because GTPases are usually active in their GTP-bound state, these proteins might act as sensors of the GTP/GDP ratio and respond under starvation conditions, when the GTP/GDP ratio is lower, by reducing mitoribosome assembly to match the reduced nutrient availability. At present, two mitochondrial GTPases (Mtg1, Mtg2) are associated with the IMM and have been reported to interact with the immature mt-LSU (Kotani et al. 2013), whereas, thus far, only one (C4orf14, or NOA1) has been shown to be involved in mt-SSU assembly (He et al. 2012).

ATP-dependent RNA helicases bind and remodel RNA possibly promoting the unwinding of RNA to initiate ribonucleoprotein (RNP) assembly or the displacement of RNA from RNP particles (Linder and Jankowsky 2011). At present, few such helicases have been identified in human mitochondria and, of these, SUPV3L1 is important in mt-RNA metabolism and for the stable maintenance of mtDNA (Borowski et al. 2013), whereas DDX28 (Tu and Barrientos 2015) and DHX30 are both involved in mitoribosome assembly (Antonicka and Shoubridge 2015).

A number of other factors have been identified as being involved in the assembly of mammalian mitoribosomes. Knockout of the gene encoding mTERF3 in mice and flies causes an increase in mtDNA transcription. Intriguingly, biogenesis of mt-LSU is also affected. RNA immunoprecipitation assays have revealed an association with 16S rRNA, consistent with mTERF3 playing a role in the assembly of the mt-LSU (Wredenberg et al. 2013). Other non-mitoribosomal proteins have been shown by RNA immunoprecipitation to bind 16S rRNA, such as a member of the Fas-activated Serine Threonine Kinase family FASTKD2 and the aforementioned helicase DDX28, both of which have been shown to be required for mt-LSU assembly (Antonicka and Shoubridge 2015; Popow et al. 2015; Tu and Barrientos 2015). Interestingly, the mAAA-protease, a membrane-spanning homo- or hetero-oligomer of two proteins AFG3L2 and paraplegin, also appears to affect mt-LSU assembly. Prior to insertion into the mt-LSU, cleavage of the subunit bL32m has been shown to be important in certain species but this does not happen in the conventional fashion. Rather than co-translocational cleavage by the matrix metalloendopeptidase, maturation occurs following complete import and is effected by the mAAA-protease (Nolden et al. 2005). The autosomal recessive spastic paraplegia type 7 can be caused by pathogenic mutations in paraplegin, the loss of which has been shown, in yeast and certain mouse tissues, to reduce the cleavage of bL32 leading to a mitoribosomal assembly defect. Similarly, defects of AFG3L2 can cause a rare form of spinocerebellar ataxia (SCA28) and tissue-specific mouse knockouts result in decreased steady state levels of bL3m (Almajan et al. 2012). C7orf30 (MALSU1) promotes the incorporation of uL14m into the mt-LSU, an event that is necessary for subunit stability (Rorbach et al. 2012; Fung et al. 2013). Depletion of the IMM protein MPV17L2, which is known to interact with the mt-LSU, leads to a reduction of both subunits, highlighting a possible role for this protein in their assembly (Dalla Rosa et al. 2014). Finally, a small proportion of GRSF1 has been suggested to be involved in the assembly of the mt-SSU, as its depletion leads to the accumulation of incomplete mt-SSU; however, this has not been experimentally determined (Antonicka et al. 2013; Jourdain et al. 2013).

## Molecular mechanisms of mitochondrial translation

**Fig. 2:**
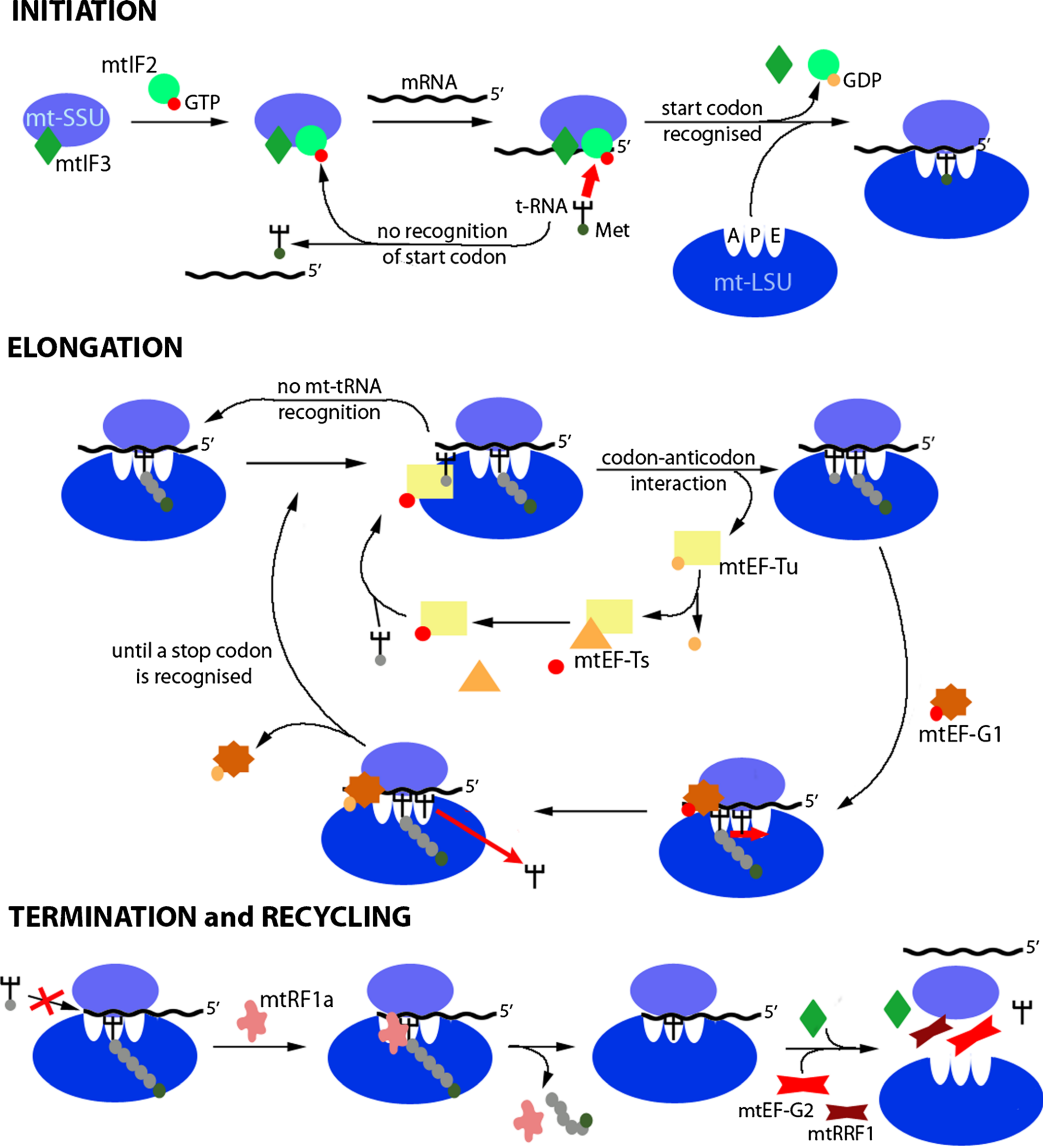
Representation of protein synthesis in human mitochondria showing the four phases of initiation, elongation, termination and recycling that comprise mitochondrial translation. Following ribosome recycling (*top*), the mitochondrial small subunit (*mt-SSU* in *blue*) remains bound to initiation factor mtIF3 (*dark green*). Initiation commences as mtIF2 (*light green*) bound to a GTP molecule (*red*) joins this complex. Once successful recruitment of mRNA has been achieved and fMet-tRNA^Met^ in the P-site anchors to the start codon, GTP is hydrolysed to GDP (*orange*), the initiation factors are released and the mitochondrial small subunit (*mt-LSU* in *darker blue*) can associate, forming the monosome. During elongation (*centre*), the nascent polypeptide chain is bound to a P-site tRNA, while the A-site is sampled by charged mt-tRNAs delivered by mitochondrial elongation factor-Tu (*mtEF-Tu* in *yellow*), until the correct codon-anticodon pair forms. GTP hydrolysis and mtEF-Tu release follows together with exchange of the GDP (*light orange*) for a new GTP molecule mediated by mtEF-Ts (*orange*). The charged A-site mt-tRNA changes its conformation juxtaposing its amino acid to that of the extending nascent chain within the peptidyl-transferase centre. This facilitates peptide bond formation transferring the polypeptide chain onto the A-site mt-tRNA. The elongation factor mtEF-G1 (*dark orange*) promotes the ribosome movement that repositions the mt-mRNA within the 55S and the mt-tRNAs from the A- and P-sites to the P- and E-sites. The E-site mt-tRNA leaves the monosome in anticipation of a new round of elongation. This cycle continues until the polypeptide is complete and a stop codon is presented in the A-site. Termination (*bottom*) described the recognition of the stop codon by a release factor protein (mtRF1a in *pink*), which then adopts a modified conformation that promotes hydrolysis of the ester bond anchoring the nascent chain to the final mt-tRNA. Once the polypeptide chain is released, the two recycling factors, mtRRF1 (*dark red*) and mtRRF2 (*red*), promote the dissociation of the ribosomal subunits and premature re-association is prevented by the formation of an mtIF3/mt-SSU complex

Transcription of human mtDNA is driven from within the noncoding region by promoters on both strands to form polycistronic transcription units (Gustafsson et al. 2016). Processing of these units is driven in part by the natural positioning and folding of mt-tRNA structures, which are excised by the tRNase Z ELAC2 mitochondrial isoform (Rossmanith 2011) and the three subunit protein-only mitochondrial RNase P (Holzmann et al. 2008). Maturation of all light-strand protein-encoding RNAs is facilitated by the addition of a poly(A) tail of approximately 50 nucleotides by the mitochondrial poly (A) polymerase (Tomecki et al. 2004, Temperley et al. 2010a, 2010b). The resultant nine monocistronic and two dicistronic mt-mRNA species are translated by the mitoribosome in a process that can be divided into initiation, elongation, termination and recycling (Fig. 2; for reviews, see Christian and Spremulli 2012; Ott et al. 2016). These mt-mRNA species have a modified codon usage. Mitochondria from different organisms vary in which codon is reassigned but an almost universal change is for the UGA stop codon to be recognised as tryptophan. Other changes include the recognition of AUA as methionine and AGA or AGG becoming unassigned codons that are not recognised by any mt-tRNA or protein factor (Chrzanowska-Lightowlers et al. 2011; Suzuki et al. 2011).

The first step in the initiation of protein synthesis is the recruitment of the mt-mRNA to the mt-SSU, which is bound by the initiation factor mtIF3 to inhibit premature re-association with the mt-LSU. The PPR protein, mS39, found at the entrance of the mt-mRNA channel has been reported to aid recruitment of the mt-mRNA (Amunts et al. 2015; Greber et al. 2015). The codons recognised as initiating triplets by the mitoribosome are AUG, AUA and AUU to which f-Met-tRNAMet (a subset of met-tRNAMet is formylated by mitochondrial methionyl-tRNA formyltransferase; Tucker et al. 2011) is recruited by mtIF2:GTP. Charged mt-tRNAMet and mtIF2 can bind the mt-SSU in the absence of the mt-mRNA; however, the association is considerably weaker. If a positive codon:anticodon interaction occurs, then a stable complex is created and the interaction with the mt-LSU follows. This formation of the monosome triggers hydrolysis of the mtIF2-bound GTP to GDP concomitantly with the release of initiation factors 2/3 from the mt-SSU. If f-Met-tRNAMet is not available or if the start codon is not present in the P-site, the inspection fails and the mRNA is released.

In mt-mRNAs, the start codon is found at or very near the 5’ terminus in monocistronic transcripts, with efficiency of translation initiation being predicted to be reduced the more distal the start codon (Christian and Spremulli 2010). The two bicistronic RNA units, *RNA7* (*MTND4/MTND4L*) and *RNA14* (*MTATP8/MTATP6*), present an enigma with respect to translation initiation. Since these transcripts contain overlapping open reading frames (ORF), the downstream ORF has, by default, a 5’-UTR that consists of the upstream coding sequence. How the mitoribosome locates this internal initiating codon is still unclear.

Once the monosome is formed, elongation of the nascent chain can start. In this step, a ternary complex forms composed of the mitochondrial elongation factor mtEF-Tu, GTP and a charged mt-tRNA, which can enter the A-site. If the correct codon:anticodon interaction takes place, the mitoribosome stimulates the hydrolysis of GTP leading to the release of GDP:mtEF-Tu. The GTP:mtEF-Tu complex is restored by the direct interaction of mtEF-Tu with the nucleotide exchange factor mtEF-Ts (Cai et al. 2000). After the release of mtEF-Tu, the formation of the peptide bond is catalysed at the peptidyl transferase centre (PTC) in the mt-LSU. Once the bond is formed, the P-site of the mitoribosome is occupied by a deacylated mt-tRNA, and the dipeptidyl-tRNA is found in the A-site. The interaction of the elongation factor mtEF-G1 with the mitoribosome alters its structural conformation, which leads to the release of the mt-tRNA from the P-site and the movement of the dipeptidyl-tRNA by three nucleotides into the P-site. Recently, the presence of the E-site in mammalian mitoribosome has been confirmed (Amunts et al. 2015; Greber et al. 2015). Although different from the characteristic bacterial E-site, the deacylated mt-tRNA moves to this site before exiting the mt-monosome. The elongation process is then reiterated until a stop codon is positioned in the A-site.

Protein synthesis is complete once a stop codon enters the A-site but the nascent peptide then needs to be released from the mitoribosome. The termination codon is recognised by a protein rather than by a tRNA species and, in human mitochondria, mitochondrial release factor 1a (mtRF1a) is believed to be sufficient to terminate all 13 ORFs (Soleimanpour-Lichaei et al. 2007). This factor is a class I RF, which, unlike class II, demonstrates sequence-specific recognition of the A-site codon (UAA, UAG). This first step in termination is mediated by the interaction of two protein domains with the RNA, the tripeptide motif and the tip of the α-5 helix. Once these two regions have recognised the A-site triplet as a stop signal, the RF structure alters to position a second conserved domain, the GGQ motif (Frolova et al. 1999) into the PTC. This facilitates the hydrolysis of the ester bond between mt-tRNA and the final amino acid. Hence, mtRF1a in the presence of GTP promotes the release of the polypeptide from the mt-LSU (Schmeing et al. 2005). In human mitochondria, UAA and UAG are used as stop codons to terminate nine monocistronic and two bicistronic ORFs, respectively. The sequencing of human mtDNA in 1981 logically suggested that, since the triplets following the coding sequence for the remaining two ORFs, *MTCO1* and *MTND6*, were AGA and AGG, respectively, these must have been recoded as alternative stop codons, as no corresponding tRNA was present in the mitochondrial genome (Anderson et al. 1981). Fine mapping of the termination codons of transcripts positioned at the A-site of the human mitoribosome in intact cells, however, showed that these two species terminated with the classical UAG codon (Temperley et al. 2010a, 2010b). An explanation for this observation is that a −1 frameshift occurs, potentially driven by structured RNA immediately downstream of the termination codons within the transcripts. Although this mechanism is plausible for humans, the frameshift alone may not create a stop codon in other vertebrates. More recently, another member of the mitochondrial translation release factor family, ICT1, has been suggested to be involved in the termination of the synthesis of COXI and ND6. As discussed above, ICT1 is a structural component of mt-LSU but is not located in the proximity of the A-site. Despite this, a limited free pool of ICT1 might have peptidyl hydrolase activity, analogous to the bacterial ArfB (YaeJ; Akabane et al. 2014). Recent studies of isolated ICT1 have confirmed its ability to hydrolyse peptidyl-tRNA on stalled ribosomes but, intriguingly, not when the RNA template extends more than 14 nucleotides past the A-site (Feaga et al. 2016). This has led to the conclusion that ICT1 is unlikely to act as a natural translation terminator in vivo, as *MTCO1* and *MTND6* mt-mRNA have 3’ extensions longer than 14 nucleotides. As four members of the mammalian mitochondrial translation release factor family have been found (Chrzanowska-Lightowlers et al. 2011), other release factors might be involved in terminating the translation of these two mt-mRNAs, perhaps in other mammalian species (Young et al. 2010).

After the release of the polypeptide, two ribosomal recycling factors, mtRRF1 and mtEF-G2, promote the dissociation of the ribosomal subunits and the release of mt-mRNA and deacylated mt-tRNA (Rorbach et al. 2008; Tsuboi et al. 2009). These two factors are finally released and the translation cycle can reinitiate.

## Regulation of mitochondrial translation

As the components of the OXPHOS complexes are synthesised both in the cytosol and in the mitochondrial matrix, their synthesis must be coordinated in order to lead to an efficient assembly of the complexes. Translational activators abound in yeast mitochondria. These proteins regulate the synthesis of various proteins and associate selectively with the (mainly 5’) UTRs of all yeast mt-mRNA species (for a review, see Herrmann et al. 2013). Their exact mode of function remains unknown but these activators establish a feedback loop whereby the absence of available partners to produce a complete OXPHOS complex can inhibit further translation of the associated transcript. The absence of UTRs in the majority of human mitochondrial transcripts would appear to preclude the functioning of such proteins. However, one translational activator has been identified in human mitochondria, namely TACO1 (Weraarpachai et al. 2009). The absence of this activator in patients with pathogenic mutations of TACO1 results in the selective loss of *MTCO1* translation, which encodes COXI of complex IV. The mechanism of action of TACO1 is not known but cannot be mediated via a 5’-UTR as no such sequence exists on *MTCO1*. It has been postulated to promote the recognition of the start codon of *MTCO1* or to stabilise the polypeptide during its synthesis. TACO1 might also interact with the translation termination factor to ensure that the nascent polypeptide is not released prior to its completion (Weraarpachai et al. 2009).

As translational activators are unlikely to work in a similar fashion to those in yeast, how can the level of mitochondrial translation be modulated in response to the import of cytosolic components of the OXPHOS complexes? An important insight into this process has been advanced by the identification of MITRAC (the mitochondrial translation regulation assembly intermediate of cytochrome *c* oxidase (Mick et al. 2012). This dynamic complex appears to connect the assembly of cytochrome *c* oxidase (COX) with the synthesis of the mitochondrially encoded COXI. The molecular mechanisms underlying this link are unclear. However, pathogenic mutations have been reported in two MITRAC components C12orf62 and MITRAC12, which function early in COX assembly, and their loss results in the inhibition of COXI synthesis (Szklarczyk et al. 2012; Weraarpachai et al. 2012). No similar complex has been reported to coordinate complex I assembly with the synthesis of mitochondrial components, although a link for the assembly of complex III has been suggested (Tucker et al. 2013).

The cellular environment has also been suggested to have an effect on mitochondrial translation. In the cytosol, microRNAs interact with the proteins AGO2 and GW182, creating a complex that is able to reduce the cytosolic translation of mRNAs (Czech and Hannon 2011). MicroRNAs and AGO2 have also been postulated to form a complex within mitochondria, where, conversely, they have been reported to enhance the translation of certain transcripts during muscle differentiation (Zhang et al. 2014). The existence of microRNAs in mitochondria, however, is still controversial, particularly as no evidence has been found for the enrichment of microRNAs reported in the first detailed analysis of the transcriptome from purified human mitochondria (Mercer et al. 2011).

Mitochondrial protein synthesis can be regulated by post-translational modifications of mitoribosomal components. These can be phosphorylated (Miller et al. 2009) or acetylated as a result of the levels of ATP, acetyl-CoA and NADH within mitochondria. A role in the association of the mt-LSU and mt-SSU might be played by the mt-specific protein mS29 (see above). This protein is able to bind GTP and is found bound to GDP in the mammalian 55S structures (Amunts et al. 2015; Greber et al. 2015). The binding affinity is higher for the mt-SSU than for the monosome (O’Brien et al. 1990; Denslow et al. 1991), suggesting a possible regulatory effect of GTP-hydrolysis on mS29 (Amunts et al. 2015). In addition, mS29 contains phosphorylation sites on its intersubunit face (Miller et al. 2008) possibly further influencing the formation of the monosome (Miller et al. 2009). In addition to this example, several other mitoribosomal proteins are reported to be modified and are found in the proximity of the subunit interface or in domains crucial for translation, such as the mRNA channel, the PTC or the PES (Miller et al. 2009). The assembly of the monosome is also promoted by the complex mTERF4-NSUN4, which interacts with the mt-SSU and supports the recruitment of the mt-LSU (Cámara et al. 2011; Metodiev et al. 2014).

## Localisation of the mitoribosome and mitochondrial translation

Mitochondrial ribosomes have evolved to translate highly hydrophobic components of the OXPHOS chain that need to be inserted into the membrane to prevent aggregation and precipitation in the matrix. As a consequence, the mitoribosome is probably anchored to the membrane in close proximity to the PES to align with the insertion machinery. This interaction has been shown in yeast in recent cryo-EM tomography studies (Pfeffer et al. 2015). Biochemical studies with the bovine mitoribosome have demonstrated that approximately 40% of mitoribosomes interact with the IMM (Li and Spremulli 2000) but the way that this interaction is mediated is still unclear. This same study has suggested that the association is mediated by electrostatic interactions between mitoribosomal proteins and the IMM and by interaction between the mitoribosome and IMM proteins.

Few proteins have been reported to interact with both the IMM and the mitoribosome. One of these is OXA1L, a polytopic membrane insertase and member of the YidC/Oxa/Alb3 family, which mediates the insertion of proteins within the IMM (Hennon et al. 2015). Its homologue in yeast has been suggested to interact with the mitoribosome (Jia et al. 2003) and the C-terminus of the human protein has been cross-linked to components of the mt-LSU, namely uL13m, bL20m, bL28m, mL48, mL49 and mL51 (Haque et al. 2010), although these are not located in close proximity to the PES. MPV17L2 is an integral membrane protein that has been co-localised with the mt-LSU on sucrose gradients, suggesting its involvement in anchoring the subunit to the IMM (Dalla Rosa et al. 2014). LetM1, another IMM protein, contains a large matrix domain and its homologue in yeast (Mdm38) has been reported to interact with the mitoribosome (Lupo et al. 2011). In vitro studies performed on LetM1 have shown its interaction with the mitoribosomal protein bL36m (Piao et al. 2009) and led the authors to propose that LetM1 acts as a membrane locator for the mitoribosome. This is an interesting proposal, although the recent cryo-EM data suggest its location is not ideal for localising the mitoribosome to the membrane or for assuring the close proximity of the PES to the membrane.

Indeed, the release of the mammalian mt-LSU structure (Greber et al. 2014a, 2010b) implicates the mt-LSU component, mL45, in membrane association. This protein is localised in close proximity to the PES, a position that is ideal to allow the rapid insertion of the newly synthesised hydrophobic proteins into the membrane. The hypothesis of the involvement of mL45 in the interaction with the membrane is strengthened by its structural homology with the IMM-interacting protein Tim44 (Handa et al. 2007) and by the confirmation of the ability of its yeast homologue Mba1 to mediate the interaction between mitoribosomes and the IMM (Ott et al. 2006; Pfeffer et al. 2015). The putative role of mL45 in anchoring the mitoribosome to the membrane is intriguing but, if it does indeed play this role, it is highly likely to share it with other anchoring sites that lie within the mitoribosome and that may interact directly with the membrane or via an integral membrane protein(s) that function as a receptor(s) for the mitoribosome.

How is the newly synthesised polypeptide correctly inserted into the IMM? Two systems have been evolutionarily conserved to mediate the integration of nascent peptides into membranes: the Sec complex and the YidC/Oxa/Alb3 insertases. To date, the major candidate that has been implicated in this process in mammals is OXA1L (see above), a member of the YidC/Oxa/Alb3 insertase family, which may play a pivotal role in this process, at least for a subset of the mitochondrially encoded proteins. OXA1L is the homologue of yeast Oxa1, which is the key component of the yeast cytochrome oxidase assembly (OXA) translocase and is involved not only in the integration of mitochondrially encoded proteins, but also in the biogenesis of some nuclear-encoded mitochondrial products (Stiller et al. 2016). Intriguingly, knockdown studies of humans cells have shown that OXA1L is necessary to obtain only a functional complex I and V and does not appear to be involved in cytochrome *c* oxidase or complex III assembly in man (Stiburek et al. 2007). MITRAC and the UQCC (ubiquinol-cytochrome *c* reductase complex chaperone) members (see above) possibly function without the aid of OXA1L to assemble complexes III and IV. One other member of the YidC/Oxa/Alb3 family that has been reported to be present in human mitochondria is the homologue of the yeast Cox18p. This promotes the insertion of the C-terminal of Cox2p in yeast but its function in mammals has not been fully characterised.

## Post-translation events

As previously mentioned, at the end of translation, the mitoribosomal subunits are separated and are recycled. However, what happens to the mt-mRNA?

After translation, the mt-mRNAs are possibly protected by interaction with RNA chaperones (e.g. LRPPRC/SLIRP, FASTKD2, FASTKD4) and reused to programme a new round of translation, although these species might be degraded in order to eliminate aberrant or damaged transcripts. The identity of the RNA-degrading apparatus in mitochondria is still under investigation. Whereas the helicase involved in RNA metabolism has been identified as SUPV3L1, the key component(s) with ribonucleolytic activity may not be completely resolved. SUPV3L1 (Minczuk et al. 2002) has been found to interact with the human polynucleotide phosphorylase (PNPase; Borowski et al. 2010), which, on depletion, causes the accumulation of mt-RNA decay intermediates and the increased stability of mitochondrial transcripts. PNPase clearly is an important factor in mitochondrial RNA degradation (Chujo et al. 2012) but most of this protein is localised in the mitochondrial intermembrane space and a role in RNA import has been suggested (Wang et al. 2010). The degradation of RNA species in mitochondria is likely to be aided by REXO2, a protein found both in the cytosol and in mitochondria. This protein is an exonuclease able to digest very short oligo RNA and might help PNPase or other enzymes to degrade and recycle unwanted RNA (Bruni et al. 2013). More recently, an endoribonuclease, LACTB2, has been identified in mammalian mitochondria. This protein has been thoroughly characterised in vitro, including high resolution structural data, but its physiological function remains unclear (Levy et al. 2016).

## Mitochondrial translation and pathologies

Mitochondrial diseases are a class of heterogeneous disorders that can be characterised by mutations of either mtDNA or nuclear DNA and that cause severe defects of OXPHOS function. Symptoms can vary from mild to profound, from highly tissue selective to multi-systemic, making clinical diagnosis challenging. Many pathogenic mtDNA deletions or point mutations in genes encoding mt-tRNA and mt-mRNA have been well documented since the first reports in 1988 (Holt et al. 1988; Wallace et al. 1988). Additionally, because of the advances in whole exome sequencing, a dramatic recent increase has been seen in the identification of novel mutations in nuclear-encoded mitochondrial proteins. As mitochondrial translation has a key role in OXPHOS, mutations in most of the components involved in the process can lead to pathologies, as shown in Table 2; a comprehensive list appears in several recent reviews (Lightowlers et al. 2015; Mayr et al. 2015; Shen et al. 2016).

**Table 2:** Human genes in which mutations have been associated with mitochondrial disease

Process	Gene product
Mitoribosome assembly	TFB1M, AFG3L2, SPG7, MTG2, DDX28, DHX30, uS7m, bS16m, mS22, bL3m, bL12m, mL44
mt-tRNA aminoacylation	AARS2, CARS2, DARS2, EARS2, FARS2, HARS2, IARS2, LARS2, MARS2, NARS2, PARS2, RARS2, SARS2, TARS2, VARS2, YARS2, GARS, KARS, QARS
mt-tRNA processing and modification	ELAC2, MRPP2, GTPBP3, MTO1, MTFMT, PNPT1, TRNT1, PUS1, TRIT1, TRMU, TRMT5, NSUN3
mt-mRNA maturation/maintenance	LRPPRC, MTPAP
Translation	EFTs, EFTu, EFG1, RMND1, MITRAC12, C12orf65, TACO1, GFM2

Regarding the protein synthesis machinery, whereas several mutations of the mt-rRNAs have been associated with disease (see http://www.mitomap.org/MITOMAP), only a few mutations of mitoribosomal proteins have been identified to date. In particular, a mutation in bS16m has been linked to hypotonia and fatal neonatal lactic acidosis (Miller et al. 2004), whereas two different mutations in mS22 result in cardiomyopathy and tubulopathy (Saada et al. 2007; Smits et al. 2011). Concerning components of the mt-LSU, a mutation in uL3m has been shown to cause cardiomyopathy (Galmiche et al. 2011), a defect also reported for a mutation of the mitochondrial-specific protein mL44 (Carroll et al. 2013). Growth retardation and neurological deterioration have been diagnosed as a consequence of a mutation in the mitoribosomal protein bL12m (Serre et al. 2013). Recently, mutations in uS7m have been discovered and linked to deafness with renal and hepatic failure (Menezes et al. 2015). Further to the role of mitochondrial ribosome protein (MRP) mutations in disease, Auwerx and colleagues have reported that genetic variation in genes encoding MRPs are linked to longevity in certain mice strains. Moreover, by depleting Mrps5 in *Caenorhabditis elegans*, they have obtained evidence suggesting that a mito-nuclear protein imbalance caused by a relative decrease in mitochondrial translation can lead to activation of a mitochondrial unfolded protein response underlying this increased longevity (Houtkooper et al. 2013).

## Concluding remarks

Progress in understanding the mechanisms underlying mammalian mitochondrial gene expression in recent years has been impressive. This has been attributable, in large part, to the production of high resolution structures of the mammalian mitoribosome and the identification of pathogenic mutations in key players via whole exome sequencing. Establishing a reconstituted in vitro translation system remains an enormous challenge (accepting the impressive initial efforts by Suzuki and colleagues; Takemoto et al. 2009). Major limitations include the isolation of sufficient quantities of mammalian mitochondria that are devoid of template mt-mRNA and mt-tRNAs to use as a substrate and also the central role played by associations with the IMM and translocon, which has yet to be characterised. Structural biology and genetics will undoubtedly further our detailed understanding, particularly regarding the assembly process of the mitoribosome, but the next step will also require the establishment of a faithful in vitro reconstituted mitochondrial translation system. We look forward to this possibility with great excitement.
